# The Cx43/p38 MAPK/Runx2 signaling pathway as a potential regulatory axis in insufficient osteogenesis associated with osteoporosis

**DOI:** 10.3389/fendo.2026.1927444

**Published:** 2026-10-08

**Authors:** Dongcheng Peng, Chuntao Liang, Yuanxin Li, Jiaqi Tan, Kailin Jiang, Zhenyu Song, Xiangyu Guo, Hongkai Wang

**Affiliations:** 1 The Second Affiliated Hospital of Guilin Medical University, Guilin, China; 2 Guilin Medical University, Guilin, China

**Keywords:** Connexin 43, DIPQUO, osteogenic differentiation, osteoporosis, p38 MAPK, RUNX2

## Abstract

**Background:**

Osteoporosis is characterized by severe osteogenic insufficiency, which severely impairs bone regeneration and repair. Connexin 43 (Cx43) participates in the modulation of osteoblast function, yet its regulatory mechanism linking osteoporosis and osteogenic deficiency through the p38 mitogen-activated protein kinase (p38 MAPK)-Runt-related transcription factor 2 (Runx2) signaling axis remains unclear. This study aimed to clarify the above regulatory relationship.

**Methods:**

We constructed an ovariectomized (OVX) mouse osteoporosis model and a Cx43-knockdown MC3T3-E1 osteoblast model. Alkaline phosphatase (ALP) and Alizarin Red S staining were performed to assess osteogenic differentiation capacity, and western blot (WB) was adopted to measure the protein expression of p38, phosphorylated p38 (p-p38) and Runx2. The p38 MAPK activator DIPQUO was utilized to explore the potential molecular mechanism. Hematoxylin-eosin (HE) staining combined with WB assays were further applied to verify the *in vivo* experimental outcomes.

**Results:**

Cx43 expression was significantly decreased in the bone tissues of OVX mice, accompanied by suppressed p38 MAPK phosphorylation and downregulated Runx2 expression. Cx43 knockdown inhibited osteogenic activity, matrix mineralization as well as the activation of the p38 MAPK-Runx2 pathway in MC3T3-E1 cells. Activation of p38 MAPK by DIPQUO recovered Runx2 expression and partially rescued osteogenic defects induced by Cx43 deficiency under *in vitro* conditions. Consistent with cellular findings, OVX osteoporotic mice showed trabecular deterioration on histological examination, accompanied by reduced p-p38 and Runx2 protein levels in bone tissue.

**Conclusions:**

Collectively, Cx43 deficiency hinders osteoblast differentiation under osteoporotic status by suppressing the p38 MAPK-Runx2 signaling cascade. Pharmacological activation of p38 MAPK by DIPQUO may partially rescue osteogenic dysfunction induced by Cx43 downregulation under *in vitro* conditions. The potential relevance of the Cx43/p38 MAPK/Runx2 signaling axis to perioperative bone repair in patients with osteoporosis remains to be validated in appropriate *in-vivo* models.

## Background

Osteoporosis is a highly prevalent metabolic bone disease characterized by reduced bone mass and deteriorated bone microarchitecture, which greatly increases fragility fracture risk (1, 2). Excessive osteoclast-dominated bone resorption was long regarded as its core pathogenesis, yet accumulating evidence confirms that impaired osteoblast-derived bone formation also drives disease initiation and progression (3–6). The molecular mechanisms of insufficient osteogenesis in osteoporosis remain poorly defined, necessitating further exploration of osteoblast-associated signaling networks.

Connexin 43 (Cx43), encoded by the *GJA1* gene, is the most abundant connexin expressed in bone cells (7). It forms intercellular channels to facilitate direct communication between adjacent osteoblasts and osteocytes, coordinating cellular responses to mechanical and hormonal stimuli (8, 9). Specific Cx43 deletion in osteoblasts or osteocytes reduces bone mass, inhibits osteogenesis and blunts bone anabolic responses to parathyroid hormone and mechanical loading (10). Both clinical specimens and osteoporotic animal models exhibit decreased skeletal Cx43 expression (11, 12); however, the precise signaling cascade linking Cx43 loss to impaired osteoblast differentiation under osteoporosis remains unclear.

The p38 mitogen-activated protein kinase (p38 MAPK) is a classic pathway governing osteoblast differentiation (13). Its activation elevates the expression and transcriptional activity of Runt-related transcription factor 2 (Runx2), the master transcription factor controlling the transcription of osteogenic genes including ALP, osteocalcin and osteopontin (14). Recent studies have reported functional crosstalk between Cx43 and p38 MAPK across multiple cell types: Cx43 acts as a scaffold protein to promote cardiomyocyte p38 MAPK activation, and Cx43 downregulation suppresses p38 phosphorylation (15). Nevertheless, whether the Cx43/p38 MAPK/Runx2 axis exists in osteoblasts and mediates osteoporosis-related osteogenic defects remains unclarified.

We hypothesized that Cx43 deficiency hinders osteoblast differentiation in osteoporosis via suppressing the p38 MAPK-Runx2 pathway. To validate this hypothesis, OVX osteoporotic mice and Cx43-knockdown MC3T3-E1 cells were constructed. ALP staining, Alizarin Red S staining and WB were used to assess osteogenic differentiation, and the p38 MAPK activator 6,8-dimethyl-3-(4-phenyl-1H-imidazol-5-yl)quinolin-2(1H)-one (DIPQUO) was utilized to determine whether p38 activation reverses Cx43 knockdown-induced osteogenic damage. Targeted activation of this pathway may provide a candidate direction for exploring interventions against osteoporosis–related osteogenic impairment and represents a potential molecular clue for studying perioperative bone repair.

## Methods

### Animal model and ovariectomy surgery

All animal experimental procedures were strictly performed in accordance with the guidelines of the Animal Care and Use Committee and were approved by the Institutional Animal Ethics Committee (Ethics Approval Number: GLMU-IACUC-202610134). Eighteen 8-week-old female C57BL/6J mice were randomly divided into the control group, Sham group and OVX group (n=6 per group). Mice in the Ctrl group received neither surgical operation nor anesthesia. All mice were maintained in an SPF facility with a 12 h light/dark cycle (22 ± 2 °C, 50 ± 10% humidity), with ad libitum access to food and water. Mice were intraperitoneally anesthetized with 400 mg/kg 5% chloral hydrate and fixed in the prone position after dorsal skin preparation and disinfection. For OVX mice, bilateral ~0.5 cm dorsal incisions were made to isolate and completely resect ovaries after ligation of the surrounding oviducts, followed by layered wound suturing. Sham mice received identical incision and tissue separation without ovarian resection. All animals were administered intramuscular penicillin (1.5 mg/kg) daily for three consecutive postoperative days for infection prophylaxis. At postoperative week 8, all mice were humanely euthanized using an overdose of chloral hydrate combined with cervical dislocation. Bilateral femurs were harvested immediately for subsequent detection. Dissected femurs were cleaned of muscle and soft tissues. Bone marrow contents were removed. Femur samples were divided: one portion was fixed and decalcified for HE staining, another portion was used for total-protein extraction for western blot analysis.

### HE staining

Isolated femoral tissues were fixed, decalcified and paraffin-embedded for slicing. Sections were baked at 62 °C for 60 min, dewaxed with xylene and gradient ethanol dehydration, sequentially stained with hematoxylin and eosin, dehydrated, cleared and mounted with neutral balsam. Histological images were captured under an optical microscope.

### Cell culture

MC3T3-E1 cells were cultured at 37 °C in a humidified incubator with 5% CO_2_ in complete α-MEM medium supplemented with 10% fetal bovine serum, 100 U/mL penicillin and 100 μg/mL streptomycin, and the medium was refreshed every 3 days. When cell confluence reached 70%-80%, the medium was replaced with osteogenic induction medium (complete α-MEM supplemented with 50 μg/mL ascorbic acid-2-phosphate and 10 mM β-glycerophosphate disodium; no dexamethasone was added in this osteogenic system). For drug-treated groups, DIPQUO was added to osteogenic induction medium at a final concentration of 10 μM. The DIPQUO stock solution was prepared in DMSO, and an equal volume of DMSO was added to the vehicle control group to maintain consistent final DMSO concentration (0.1 % v/v) among all groups. Osteogenic induction medium (with identical DIPQUO or DMSO dosage) was renewed every 3 days.

### Lentivirus-mediated stable Cx43 knockdown

MC3T3-E1 cells were seeded in 6-well plates at 2×10^5^ cells/well. Upon 70%–80% confluence, cells were infected with Cx43-targeting shRNA lentivirus or negative control lentivirus diluted 1:50 in polybrene-containing α-MEM at a multiplicity of infection (MOI) of 100. The targeting sequence for Cx43 shRNA was GGGTCCTTCAGATCATATTCG; the shDNA template sense-strand sequence was 5’-GATCCGGGTCCTTCAGATCATATTCGTTCAAGAGACGAATATGATCTGAAGGACCCTTTTTTG-3’, and the antisense-strand sequence was 5’-AATTCAAAAAAGGGTCCTTCAGATCATATTCGTCTCTTGAACGAATATGATCTGAAGGACCCG-3’. After 24 h incubation, medium was replaced. Transfection efficiency was observed via fluorescence microscopy within 48–96 h, and cells were continuously screened with 3 μg/mL puromycin for four days to obtain stable Cx43-knockdown cell lines.

### Osteogenic differentiation induction

Cells were seeded in 24-well plates at 5×10^4^ cells/well, and osteogenic induction was initiated when cell density exceeded 80%. ALP activity was detected at differentiation day 7, and mineralized nodule formation was detected at differentiation day 21.

### CCK-8 cell viability assay

Cells were seeded in 96-well plates at 5×10³ cells/well and cultured overnight. After 48 h of corresponding treatment, 10 μL CCK-8 reagent was added per well followed by 2 h dark incubation at 37 °C, and absorbance at 450 nm was measured with a microplate reader.

### ALP staining

At differentiation day 7, cells were fixed with 4% paraformaldehyde for 30 min and stained with BCIP/NBT working solution for 30 min. Morphological observation was performed under light microscopy, and absorbance at 405 nm was quantified to calculate ALP activity.

### Alizarin Red S staining

At osteogenic induction day 21, cells were fixed with 4% paraformaldehyde for 30 min and stained with Alizarin Red S according to manufacturer protocols. Staining images were photographed, and mineralized area was quantified via ImageJ software.

### WB analysis

Total protein was extracted directly from fully pre–ground marrow-depleted femoral bone tissues, while cell samples were harvested after 48 h of osteogenic induction combined with DIPQUO (DIP) or equal-volume DMSO vehicle treatment. Both types of samples were lysed with RIPA lysis buffer supplemented with 1:100 protease inhibitor cocktail. The protein concentration of each sample was determined using a BCA protein quantification kit. Equal amounts of protein were separated by SDS-PAGE and transferred to PVDF membranes. The membranes were blocked with 5 % BSA-TBST for 2 h at room temperature, and then incubated overnight at 4 °C with primary antibodies: anti-β-actin (Santa Cruz, cat. No. sc-517582, 1:2000), anti-Runx2 (Beyotime Biotech Inc, cat. No. AF2593, 1:2000), anti-total p38 MAPK (Proteintech, cat. No. 80821-2-RR, 1:1000), anti-p-p38 MAPK (Beyotime Biotech Inc, cat. No. AF5887, 1:1000), anti-JNK MAPK (Proteintech, cat. No. 81629-1-RR, 1:2000), anti-p-JNK MAPK (Beyotime Biotech Inc, cat. No. AF5860, 1:2000), and anti-Cx43 (Proteintech, cat. No. 80543-1-RR, 1:4000). After thorough washing with TBST, membranes were incubated with HRP-conjugated secondary antibody (BOSTER, cat. No. BA1054, 1:5000) for 1 h at room temperature. Protein band images were acquired by chemiluminescence after additional TBST washing. Band gray values were semi-quantified using ImageJ software, and the expression levels of target proteins were normalized to β-actin. For phosphorylated proteins, signal intensities were normalized to their corresponding total protein levels: p-p38/total p38 and p-JNK/total JNK, respectively. Total protein signals were further normalized to β-actin. For quantitative analysis shown in **Figure 1C**, signal intensities of Ctrl, Sham and OVX bone samples were normalized to those of the Ctrl animal group, whose value was set as 1.

**Figure 1:**
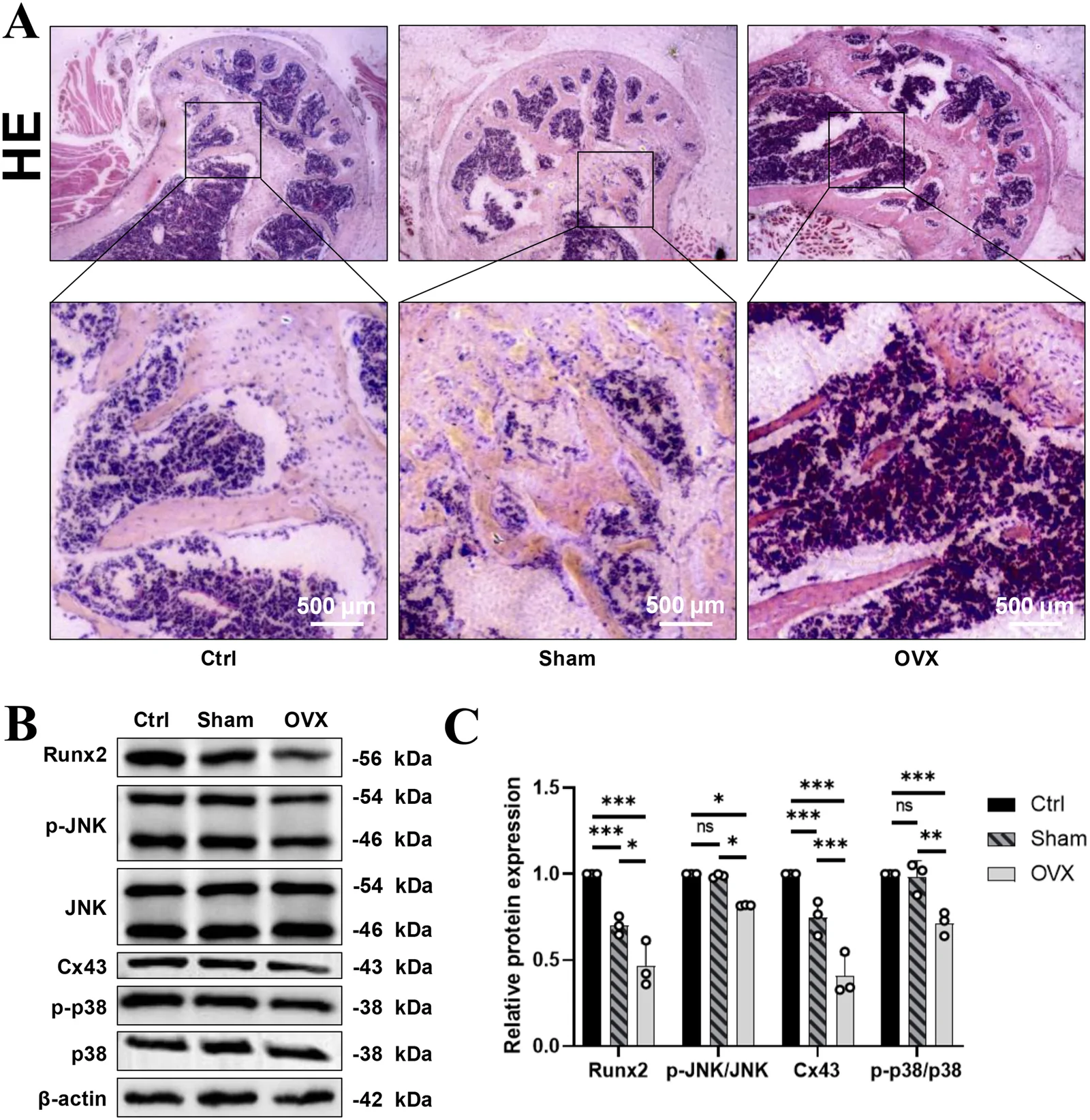
OVX surgery deteriorates bone histology and suppresses Cx43-p38/JNK-Runx2 signaling in mouse bone tissue. **(A)** Histological staining of bone from Ctrl, Sham and OVX groups; lower panels show magnified views of boxed regions. (scale bar = 500 μm). **(B, C)** Immunoblot images and quantification of Runx2, Cx43, p-JNK/JNK and p-p38/p38. In panel **(C)**, relative protein levels for Ctrl, Sham and OVX were normalized to the Ctrl animal group (Ctrl value set to 1). Statistical comparisons were performed between Ctrl vs. Sham, Ctrl vs. OVX, and Sham vs. OVX. Significance symbols above bars refer to comparisons against Ctrl, while horizontal brackets denote comparisons between Sham and OVX. Data are presented as mean ± SD, *P < 0.05, **P < 0.01, ***P < 0.001, ns, not significant. Ctrl: untreated normal animal control; Sham: sham-operated animal group; OVX: ovariectomy-induced osteoporosis animal group.

### Statistical analysis

All cellular experiments were independently repeated for at least three biological replicates. Each biological replicate contained 2-3 technical replicates. For animal experiments, n = 6 mice per group. Quantitative data were expressed as mean ± SD. Unpaired t-test was used for two-group comparisons, and one-way ANOVA followed by Tukey’s post-hoc test was applied for multiple-group comparisons. P < 0.05 was considered statistically significant.

## Results

### Cx43 is downregulated in osteoporotic bone tissue

Cx43 expression was detected in bone tissues from Ctrl, Sham and OVX mice. HE staining (**Figure 1A**) revealed fewer and thinner trabeculae with widened inter-trabecular space in OVX mice compared with both Ctrl and Sham groups, suggesting trabecular bone deterioration after OVX surgery (**Figures 1B, C**). For the quantitative analysis in **Figure 1C**, protein levels of Sham and OVX groups were normalized to the Ctrl animal group (set as 1). WB quantification revealed significantly lower Cx43 expression in the OVX group relative to the Ctrl group (P < 0.001); Cx43 level in the Sham group was comparable to that of Ctrl. Meanwhile, compared with Ctrl animals, OVX bone displayed reduced p38 MAPK phosphorylation (P < 0.01) and decreased Runx2 protein abundance (P < 0.05), whereas no significant differences were observed between Sham and Ctrl. As classic MAPK subtypes, p38 exhibited a more obvious activity decline than JNK in osteoporotic OVX bone. Collectively, reduced skeletal Cx43 expression was accompanied by reduced p38 MAPK activation and Runx2 expression under osteoporosis. In our dataset, the Cx43-p38 axis is a relatively prominent regulatory component among the examined MAPK-related molecules in osteoporotic bone.

### Cx43 knockdown suppresses MC3T3-E1 osteogenic differentiation

Stable Cx43-knockdown MC3T3-E1 cells were constructed via lentivirus transfection (**Figure 2A**). WB confirmed remarkable Cx43 downregulation in sh-Cx43 cells (P < 0.001, **Figures 2B, C**). Cx43 knockdown significantly reduced p38 phosphorylation (P < 0.05) and Runx2 expression (P < 0.05, **Figures 2D, E**). Functional detection showed decreased ALP activity at day 7 (P < 0.05, **Figures 2F, G**) and impaired mineralized nodule formation at day 21 (P < 0.01, **Figures 2H, I**) after Cx43 depletion.

**Figure 2:**
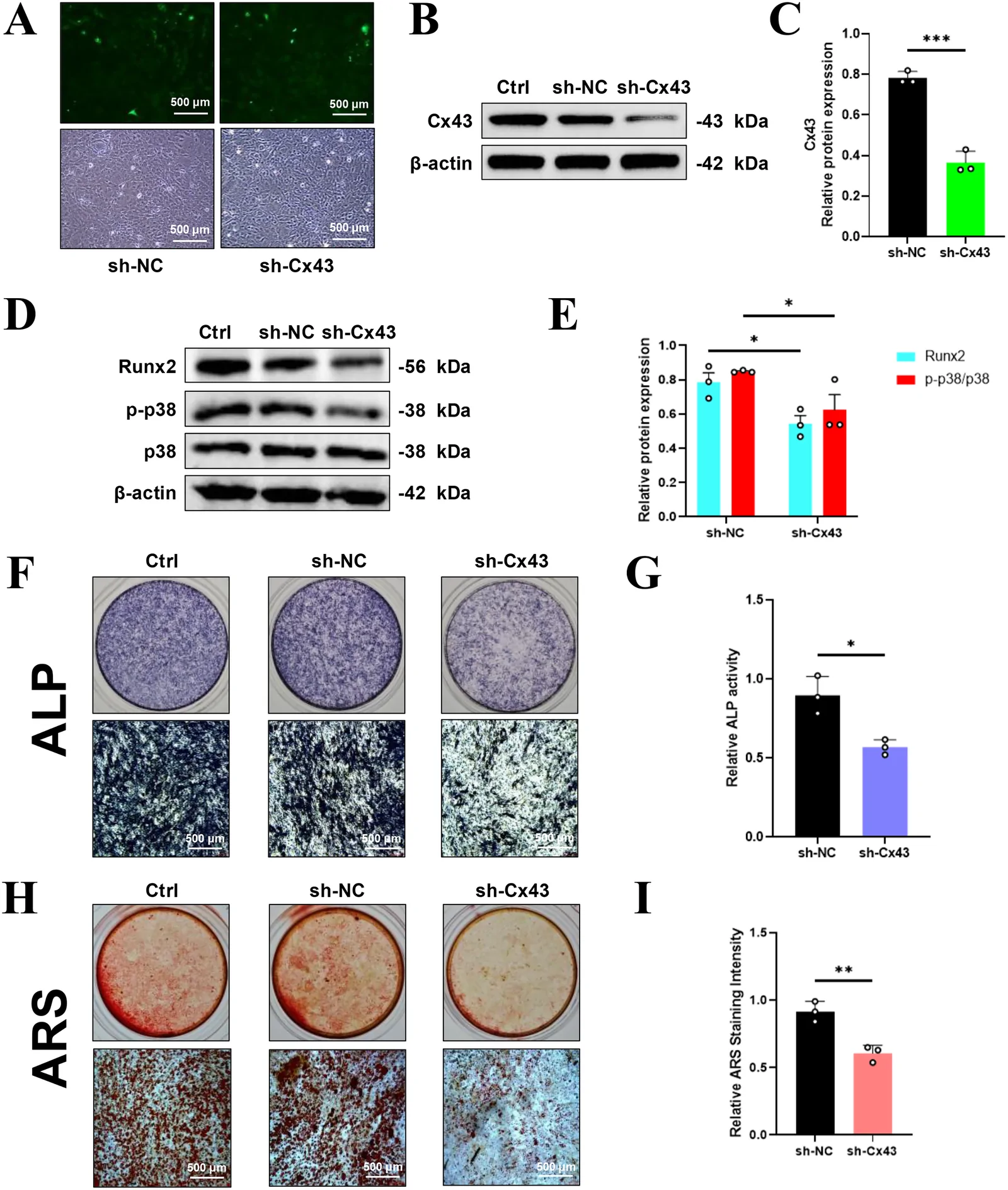
Cx43 knockdown inhibits osteogenic differentiation and blocks p38 pathway in osteoblasts. **(A)** Fluorescence microscopy images of sh-NC and sh-Cx43 cells. (scale bar = 500 μm). **(B, C)** Western blot and statistics confirming Cx43 knockdown efficiency. **(D, E)** Immunoblot quantification for Runx2 and p-p38/p38. **(F, G)** ALP staining and relative activity analysis. (scale bar = 500 μm) **(H, I)** ARS mineralized nodule staining and semi-quantitative mineralization detection. Data are presented as mean ± SD; ns, not significant; *P < 0.05, **P < 0.01, ***P < 0.001 vs. sh-NC. sh-NC: negative control; sh-Cx43: Cx43 silenced group. (scale bar = 500 μm).

### DIPQUO activates p38 MAPK and improves physiological osteogenic differentiation

Dose-gradient DIPQUO (0, 1, 5, 10, 20 μM) was applied to untreated MC3T3-E1 cells for 48 h. CCK-8 data showed no obvious cytotoxicity below 10 μM, while 20 μM DIPQUO slightly inhibited cell viability (P < 0.05, **Figure 3A**); thus 10 μM was selected for subsequent trials. WB demonstrated that 10 μM DIPQUO elevated p38 phosphorylation (P < 0.05) and Runx2 expression (P < 0.05, **Figures 3B, C**), compared with the vehicle control. Consistently, DIPQUO treatment increased ALP activity on day 7 (P < 0.01, **Figures 3D, E**) and mineralized nodule formation on day 21 (P < 0.001, **Figures 3F, G**). *In vitro* data verified DIPQUO activates the p38 MAPK-Runx2 axis and facilitates normal osteogenic differentiation.

**Figure 3:**
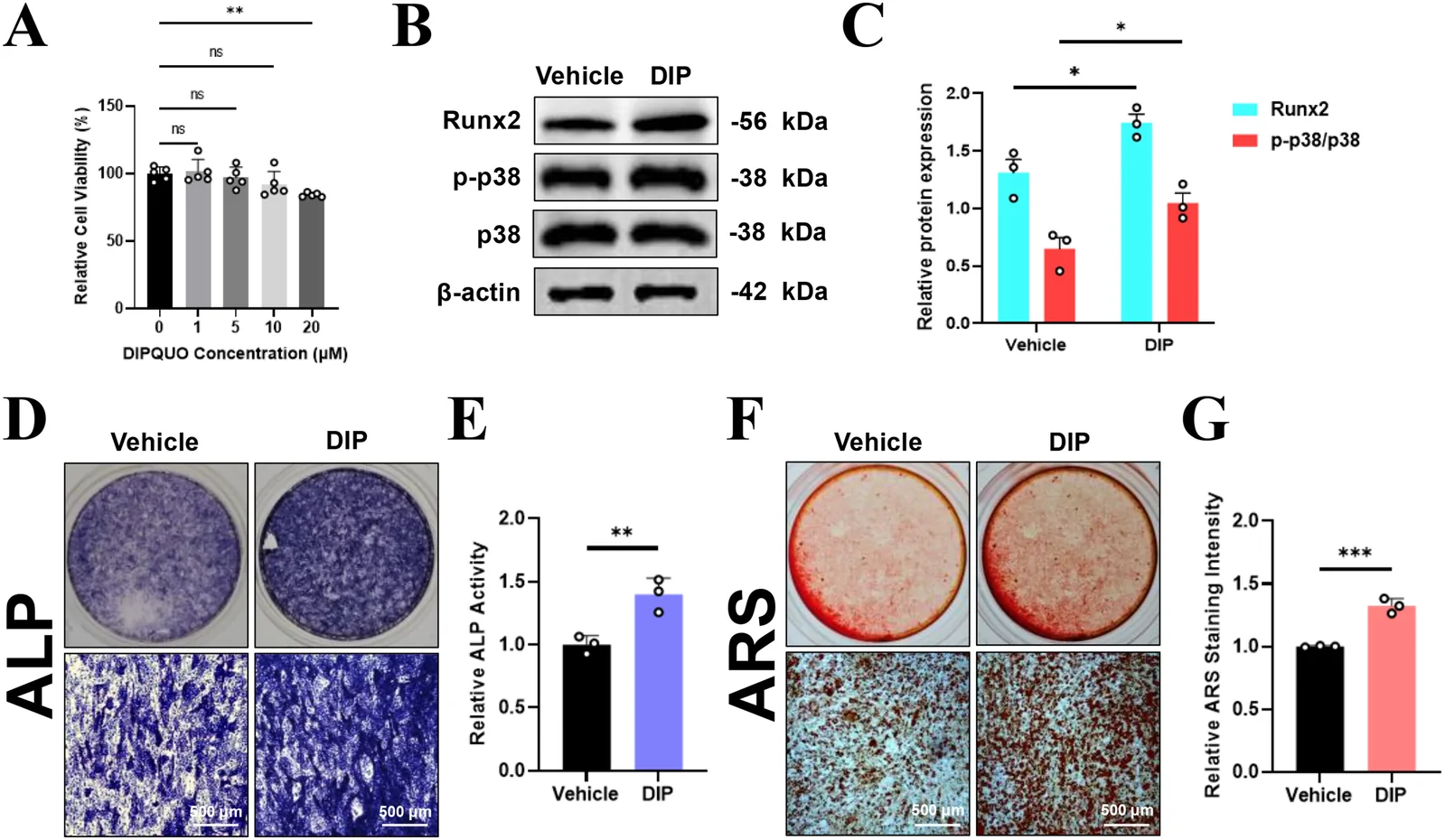
DIPQUO activates p38 signaling and facilitates osteoblast differentiation *in vitro*. **(A)** CCK-8 cell viability after gradient DIPQUO treatment. **(B, C)** Western blot and quantification of Runx2 and p-p38/p38. **(D, E)** ALP staining and quantitative activity measurement. (scale bar = 500 μm). **(F, G)** ARS staining and semi-quantitative mineralization analysis. Data are presented as mean ± SD; ns, not significant; *P < 0.05, **P < 0.01, ***P < 0.001 vs. vehicle control. Vehicle control: DMSO-treated control; DIP: DIPQUO-treated group. (scale bar = 500 μm).

### DIPQUO-mediated p38 activation rescues Cx43 knockdown-induced osteogenic defects

DIPQUO supplementation restored p38 phosphorylation and Runx2 expression in Cx43-deficient osteoblasts (P < 0.05, **Figures 4A, B**). Functional staining further validated that DIPQUO significantly rescued declined ALP activity and impaired matrix mineralization caused by Cx43 knockdown (P < 0.05 vs sh-Cx43 group, **Figures 4C–F**).

**Figure 4:**
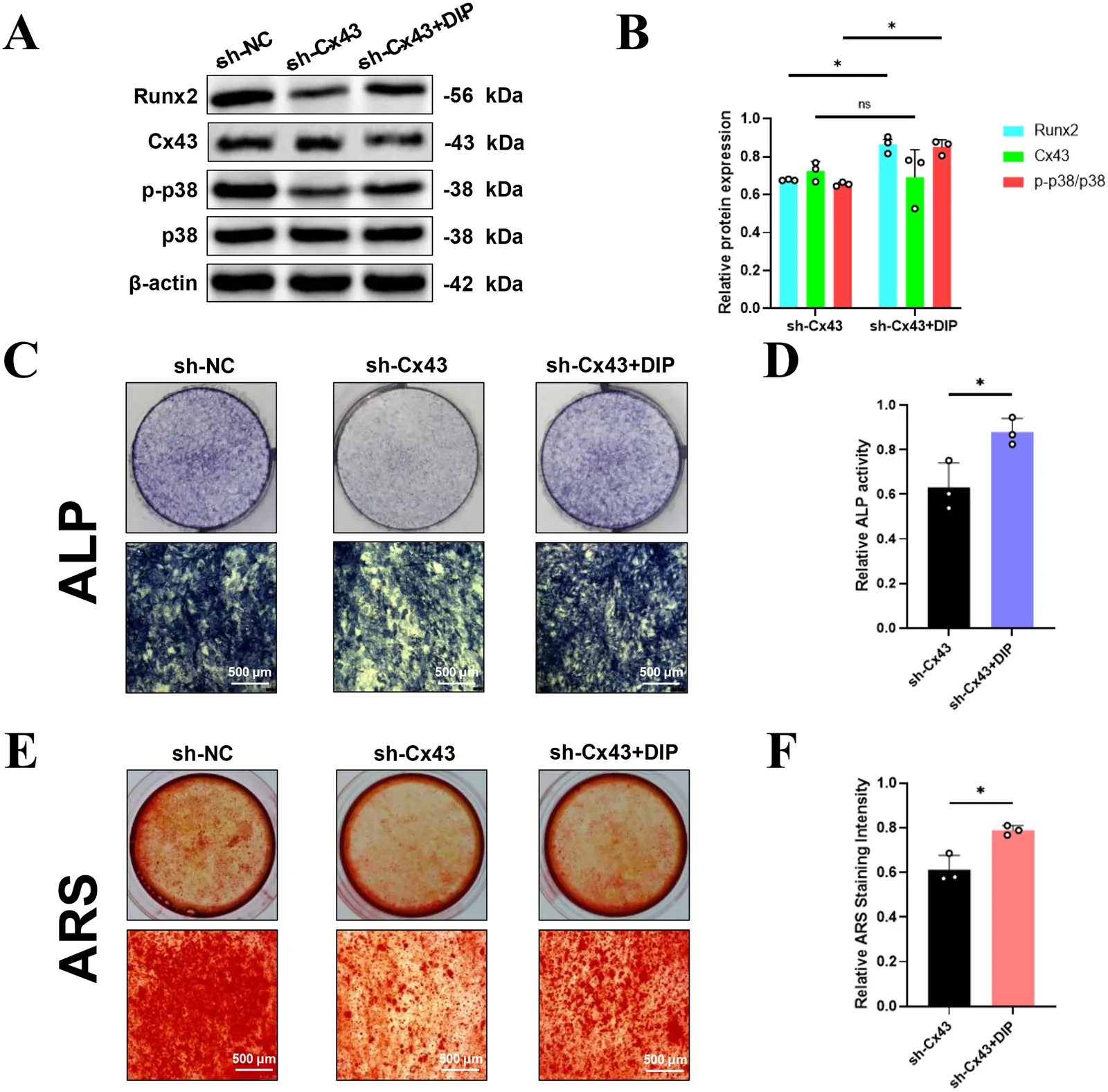
DIPQUO rescues Cx43 depletion-caused osteogenic suppression via recovering p38/Runx2 signaling. **(A, B)** Western blot and quantitative detection of Runx2, Cx43 and p-p38/p38 among sh-NC, sh-Cx43 and sh-Cx43+DIP groups. **(C, D)** ALP staining and relative activity statistics. (scale bar = 500 μm). **(E, F)** ARS staining and semi-quantitative mineralization evaluation. Data are presented as mean ± SD; ns, not significant; *P < 0.05, **P < 0.01 vs. indicated group. sh-NC: negative control; sh-Cx43: Cx43 knockdown; sh-Cx43+DIP: Cx43-silenced cells treated with DIPQUO. (scale bar = 500 μm).

## Discussion

Osteoporosis is a prevalent metabolic bone disease with reduced bone mass and elevated fracture risk, imposing heavy socioeconomic burdens worldwide (16). Osteoporotic patients commonly require orthopedic surgeries including internal fixation, joint replacement and spinal fusion, yet poor bone regeneration leads to delayed healing, implant loosening and higher revision rates (17–19). Hence, exploring osteogenic regulatory mechanisms and developing novel perioperative pro-osteogenic strategies bears prominent clinical value. Our study identifies the Cx43/p38 MAPK/Runx2 axis as a new regulatory pathway for osteoblast differentiation: Cx43 insufficiency restrains p38 MAPK activity, downregulates Runx2 and blocks osteogenic maturation, whereas p38 activation induced by DIPQUO could partially rescue osteogenic damage under *in vitro* conditions, providing preliminary clues for exploring potential targets for perioperative bone repair.

As the predominant gap junction protein in bone encoded by *GJA1*, Cx43 is indispensable for skeletal development and bone homeostasis (7). Cell-specific Cx43 knockout leads to osteopenia and blunted bone anabolic responses to mechanical loading and parathyroid hormone (20). Consistent with published data, OVX mice exhibited decreased skeletal Cx43 accompanied by deteriorated trabecular microstructure; *in vitro* Cx43 knockdown suppressed ALP activity, mineralization and Runx2 expression. Low skeletal Cx43 expression may be one factor contributing to impaired bone regeneration in osteoporotic conditions.

The p38 MAPK cascade controls osteogenic differentiation via modulating Runx2 transcription (21). Existing literature has confirmed Cx43 positively regulates p38 phosphorylation in cardiomyocytes (22, 23), and our study extends this regulatory mode to osteoblasts: Cx43 depletion reduces p38 phosphorylation without changing total p38 protein level, accompanied by Runx2 downregulation both *in vivo* and *in vitro*, supporting a potential upstream regulatory role of Cx43 for the p38 MAPK-Runx2 axis in bone-forming cells.

Most importantly, DIPQUO-induced p38 activation effectively offsets osteogenic defects caused by Cx43 loss (24), indicating direct p38 activation bypasses upstream Cx43 deficiency to promote osteoblast maturation, providing preliminary *in vitro* experimental clues for future exploration of local perioperative drug application. Considering DIPQUO preferentially targets p38 β, we interpret these phenotypes based on convergent *in vitro* readouts, while potential off-target effects on other MAPK members cannot be fully excluded; future work should test local delivery in osteoporotic fracture or bone-defect animal models.

Current clinical bone repair approaches including autologous/allogeneic bone graft and synthetic substitutes have inherent drawbacks such as donor injury and unstable osteoinduction (25–27); systemic teriparatide is limited by frequent injection and side effects for short-term perioperative use (28). In contrast, small-molecule DIPQUO can be locally delivered via graft material mixing, implant coating or injectable hydrogel, with low cost and favorable chemical stability, which may offer potential directions for future translational exploration (24).

Notably, Cx43 exerts divergent regulatory effects on p38 MAPK across distinct cell types: Cx43 overexpression inhibits p38 activity in multiple tumor cells but positively regulates p38 in osteoblasts and ligament fibroblasts, suggesting cell microenvironment determines Cx43 downstream signaling preference (29, 30). Our study clarifies the positive Cx43-p38 regulatory relationship specifically in osteoblasts.

This study has several limitations. First, DIPQUO’s *in vivo* perioperative therapeutic effect needs verification in osteoporotic fracture/fusion animal models. Second, this study only adopted the p38 MAPK agonist DIPQUO for gain-of-function rescue assays. We did not perform pharmacological inhibition experiments using SB203580 to block p38 activity, which cannot fully exclude potential off-target effects of DIPQUO. Further loss-of-function verification is required to definitively confirm that the osteoprotective effect of DIPQUO is strictly p38 MAPK-dependent. Moreover, Runx2 loss-of-function assays would further confirm whether p38-mediated osteogenic rescue is Runx2-dependent, which merits further investigation in future work. Third, systematic preclinical safety and dosage screening of DIPQUO are required before clinical transformation. Only one shRNA construct was used for Cx43 knockdown in MC3T3-E1 cells, and off-target interference cannot be fully excluded; multiple independent shRNAs would strengthen these observations in further work. Fourth, our OVX-model characterization depends on qualitative HE trabecular histology without Micro-CT-derived quantitative bone microarchitecture parameters (e.g., BV/TV, Tb.N, Tb.Th, Tb.Sp, BMD). Although OVX is a well-recognized pre-clinical model for post-menopausal osteoporosis, the absence of such quantitative metrics is a limitation of this work. Future *in-vivo* studies will include Micro-CT for thorough bone-phenotype assessment.

## Conclusions

In summary, osteoporotic Cx43 downregulation inhibits osteoblast differentiation via suppressing the p38 MAPK-Runx2 pathway, and small-molecule DIPQUO-mediated p38 activation could partially rescue *in vitro* Cx43 deficiency-induced osteogenic dysfunction. The Cx43/p38 MAPK/Runx2 signaling axis may contribute to normal osteoblast function; whether local surgical-site p38 activation could serve as a prospective strategy to improve postoperative bone healing and surgical prognosis for osteoporotic patients remains to be investigated in further *in-vivo* experiments. Further *in vivo* trials using clinically relevant animal models are required before any clinical translation for perioperative bone repair can be considered.

## Data Availability

The raw data supporting the conclusions of this article will be made available by the authors, without undue reservation.

## References

[1] Chin KY Ng BN Rostam MKI Muhammad Fadzil NFD Raman V Mohamed Yunus F . A mini review on osteoporosis: From biology to pharmacological management of bone loss. J Clin Med. (2022) 11:6434. doi: 10.3390/jcm11216434 36362662 PMC9657533

[2] Ali D Tencerova M Figeac F Kassem M Jafari A . The pathophysiology of osteoporosis in obesity and type 2 diabetes in aging women and men: The mechanisms and roles of increased bone marrow adiposity. Front Endocrinol (Lausanne). (2022) 13:981487. doi: 10.3389/fendo.2022.981487 36187112 PMC9520254

[3] Yao H Cui Y Li P Sun S Peng C Wu D . The role and mechanisms of bone microenvironment regulators in osteoporosis: Novel intervention strategies for addressing the challenges of aging. Front Endocrinol (Lausanne). (2026) 17:1783422. doi: 10.3389/fendo.2026.1783422 41852468 PMC12991982

[4] Liang B Burley G Lin S Shi YC . Osteoporosis pathogenesis and treatment: Existing and emerging avenues. Cell Mol Biol Lett. (2022) 27:72. doi: 10.1186/s11658-022-00371-3 36058940 PMC9441049

[5] Zhivodernikov IV Kirichenko TV Markina YV Postnov AY Markin AM . Molecular and cellular mechanisms of osteoporosis. Int J Mol Sci. (2023) 24:15772. doi: 10.3390/ijms242115772 37958752 PMC10648156

[6] Liu J Zhang X Hou H Ouyang J Dai J . Advances in osteoblast and mitochondrial dynamics and their transfer in osteoporosis. J Cell Mol Med. (2024) 28:e70299. doi: 10.1111/jcmm.70299 39700051 PMC11657594

[7] Stains JP Watkins MP Grimston SK Hebert C Civitelli R . Molecular mechanisms of osteoblast/osteocyte regulation by connexin43. Calcif Tissue Int. (2014) 94:55–67. doi: 10.1007/s00223-013-9742-6 23754488 PMC3815501

[8] Zhao D Wu J Acosta FM Xu H Jiang JX . Connexin 43 hemichannels and prostaglandin E(2) release in anabolic function of the skeletal tissue to mechanical stimulation. Front Cell Dev Biol. (2023) 11:1151838. doi: 10.3389/fcell.2023.1151838 37123401 PMC10133519

[9] Li G Qi M Wang Y Liang S Xu H . Molecular communication from bone to skeletal muscle: An overview. Front Cell Dev Biol. (2025) 13:1715009. doi: 10.3389/fcell.2025.1715009 41278197 PMC12634512

[10] Hashida Y Nakahama K Shimizu K Akiyama M Harada K Morita I . Communication-dependent mineralization of osteoblasts via gap junctions. Bone. (2014) 61:19–26. doi: 10.1016/j.bone.2013.12.031 24389413

[11] Ma L Hua R Tian Y Cheng H Fajardo RJ Pearson JJ . Connexin 43 hemichannels protect bone loss during estrogen deficiency. Bone Res. (2019) 7:11. doi: 10.1038/s41413-019-0050-2 31016065 PMC6476886

[12] Yang L Zhou G Li M Li Y Yang L Fu Q . High glucose downregulates connexin 43 expression and its gap junction and hemichannel function in osteocyte-like Mlo-Y4 cells through activation of the P38mapk/Erk signal pathway. Diabetes Metab Syndr Obes. (2020) 13:545–57. doi: 10.2147/dmso.S239892 32161481 PMC7049751

[13] Eapen A Sundivakkam P Song Y Ravindran S Ramachandran A Tiruppathi C . Calcium-mediated stress kinase activation by Dmp1 promotes osteoblast differentiation. J Biol Chem. (2010) 285:36339–51. doi: 10.1074/jbc.M110.145607 20841352 PMC2978562

[14] Lee CH Huang YL Liao JF Chiou WF . Ugonin K promotes osteoblastic differentiation and mineralization by activation of P38 Mapk- and Erk-mediated expression of Runx2 and Osterix. Eur J Pharmacol. (2011) 668:383–9. doi: 10.1016/j.ejphar.2011.06.059 21806985

[15] Wu L Jiang T Fu Z Wang L You H Xue J . Connexin 43 dephosphorylation at serine 282 induces spontaneous arrhythmia and increases susceptibility to ischemia/reperfusion injury. Heliyon. (2023) 9:e15879. doi: 10.1016/j.heliyon.2023.e15879 37215881 PMC10196788

[16] Ding Q Zhou B Leung J Kwok T Su Y . Global trends in burden of fractures, low bone mineral density, and high body mass index from 1990 to 2021, an age-period-cohort analysis. Osteoporos Int. (2025) 36:1855–68. doi: 10.1007/s00198-025-07570-6 40526145

[17] Harris AB Lantieri MA Agarwal AR Golladay GJ Thakkar SC . Osteoporosis and total knee arthroplasty: Higher 5-year implant-related complications. J Arthroplasty. (2024) 39:948–953.e1. doi: 10.1016/j.arth.2023.10.045 37914037

[18] Hejrati N Martens B Jost B Bozinov O Stienen MN . Failure of an expandable lumbar interbody spacer - a critical analysis of secondary collapse, pseudoarthrosis and revision rates after thoracolumbar fusion. Eur Spine J. (2025) 34:182–90. doi: 10.1007/s00586-024-08539-5 39455434

[19] Lu X Zhang A Zhang C Guo D Li ZA Xie D . Targeting the osteoporotic bone microenvironment: Mechanistic insight and therapeutic biomaterials for accelerating bone regeneration. Bioact Mater. (2026) 62:96–122. doi: 10.1016/j.bioactmat.2026.02.024 41853699 PMC12993006

[20] Pacheco-Costa R Davis HM Sorenson C Hon MC Hassan I Reginato RD . Defective cancellous *bone* structure and abnormal response to Pth in cortical *bone* of mice lacking Cx43 cytoplasmic C-terminus domain. Bone. (2015) 81:632–43. doi: 10.1016/j.bone.2015.09.011 26409319 PMC4640960

[21] Greenblatt MB Shim JH Zou W Sitara D Schweitzer M Hu D . The P38 Mapk pathway is essential for skeletogenesis and bone homeostasis in mice. J Clin Invest. (2010) 120:2457–73. doi: 10.1172/jci42285 20551513 PMC2898605

[22] Yang Y Yan X Xue J Zheng Y Chen M Sun Z . Connexin43 dephosphorylation at serine 282 is associated with connexin43-mediated cardiomyocyte apoptosis. Cell Death Differ. (2019) 26:1332–45. doi: 10.1038/s41418-019-0277-x 30770876 PMC6748098

[23] Matsuyama D Kawahara K . Proliferation of neonatal cardiomyocytes by connexin43 knockdown via synergistic inactivation of P38 Mapk and increased expression of Fgf1. Basic Res Cardiol. (2009) 104:631–42. doi: 10.1007/s00395-009-0029-z 19377854

[24] Cook B Rafiq R Lee H Banks KM El-Debs M Chiaravalli J . Discovery of a small molecule promoting mouse and human osteoblast differentiation via activation of P38 Mapk-B. Cell Chem Biol. (2019) 26:926–935.e6. doi: 10.1016/j.chembiol.2019.03.009 31031140 PMC6642001

[25] Sakdejayont S Chobpenthai T Suksirivecharuk P Ninatkiattikul IF Poosiripinyo T . A review on bone tumor management: Cutting-edge strategies in bone grafting, bone graft substitute, and growth factors for defect reconstruction. Orthop Res Rev. (2025) 17:175–88. doi: 10.2147/orr.S521832 40357445 PMC12067467

[26] Yu D Shen W Dai J Zhu H . Treatment of large bone defects in load-bearing bone: Traditional and novel bone grafts. J Zhejiang Univ-Sci B. (2025) 26:421–47. doi: 10.1631/jzus.B2300669 40436640 PMC12119185

[27] Rodham PL Giannoudis VP Kanakaris NK Giannoudis PV . Biological aspects to enhance fracture healing. EFORT Open Rev. (2023) 8:264–82. doi: 10.1530/eor-23-0047 37158338 PMC10233810

[28] Arthur Vithran DT Essien AE Rahmati M Opoku M Keon Yon D López Sánchez GF . Teriparatide in postmenopausal osteoporosis: Uncovering novel insights into efficacy and safety compared to other treatments - a systematic review and meta-analysis. EFORT Open Rev. (2024) 9:845–61. doi: 10.1530/eor-23-0205 39222329 PMC11457814

[29] Chen D Liu Y Yang H Chen D Zhang X Fermandes JC . Connexin 43 promotes ossification of the posterior longitudinal ligament through activation of the Erk1/2 and P38 Mapk pathways. Cell Tissue Res. (2016) 363:765–73. doi: 10.1007/s00441-015-2277-6 26334722

[30] Latchford LP Perez LS Conage-Pough JE Turk R Cusimano MA Vargas VI . Differential substrate specificity of Erk, Jnk, and P38 Map kinases toward connexin 43. bioRxiv. (2024). doi: 10.1101/2023.12.30.573692 39798878 PMC11870265

